# The *Pixel Anomaly Detection Tool*: a user-friendly GUI for classifying detector frames using machine-learning approaches

**DOI:** 10.1107/S1600576724000116

**Published:** 2024-02-12

**Authors:** Gihan Ketawala, Caitlin M. Reiter, Petra Fromme, Sabine Botha

**Affiliations:** a Biodesign Center for Applied Structural Discovery, Arizona State University, Tempe, AZ 85287-5001, USA; b School of Molecular Sciences, Arizona State University, Tempe, AZ 85287-1604, USA; c NSF BioXFEL Science and Technology Center Summer Internship Program, NY 14203, USA; d Department of Physics, Arizona State University, Tempe, AZ 85287-1504, USA; The University of Western Australia, Australia

**Keywords:** machine learning, serial crystallography, image classification, X-ray free electron lasers, graphical user interfaces, X-ray diffraction patterns, data analysis, experimental artefacts

## Abstract

Data collection at X-ray free electron lasers has particular experimental challenges, such as continuous sample delivery or the use of novel ultrafast high-dynamic-range gain-switching X-ray detectors. This can result in a multitude of data artefacts, which can be detrimental to accurately determining structure-factor amplitudes for serial crystallography or single-particle imaging experiments. Here, a new data-classification tool is reported that offers a variety of machine-learning algorithms to sort data trained either on manual data sorting by the user or by profile fitting the intensity distribution on the detector based on the experiment. This is integrated into an easy-to-use graphical user interface, specifically designed to support the detectors, file formats and software available at most X-ray free electron laser facilities. The highly modular design makes the tool easily expandable to comply with other X-ray sources and detectors, and the supervised learning approach enables even the novice user to sort data containing unwanted artefacts or perform routine data-analysis tasks such as hit finding during an experiment, without needing to write code.

## Abstract 2

A novel user-friendly highly modular graphical user interface based tool is reported for sorting X-ray diffraction patterns by implementing machine learning. In a supervised learning approach, the tool can easily be used to identify and sort data containing specific experimental artefacts, or trained to perform routine data-analysis tasks such as hit finding.

## 1. Introduction

Over a decade ago, scientists first recognized the potential of exploiting the unique capabilities of X-ray free electron lasers (XFELs) for the structure solution of biological macromolecules (Chapman *et al.*, 2011 ▸; Boutet *et al.*, 2012 ▸). Using the ultrashort ultrabright X-ray pulses generated by these novel sources, the diffraction pattern of micrometre-sized crystals, or even single biological particles, can be recorded before the sample is ultimately plasmarized by the interacting X-ray beam, in an approach termed ‘diffraction before destruction’ (Neutze *et al.*, 2000 ▸; Barty *et al.*, 2012 ▸). Using a variety of sample-delivery techniques [see *e.g.* Barends *et al.* (2022 ▸)], millions of single crystals or particles are streamed across the X-ray path in random orientations, where they interact with the ultrashort ultrabright X-ray pulses. While the brilliance of a single X-ray pulse obliterates the sample, the femtosecond pulse duration enables the diffraction pattern to be collected before structure-altering radiation damage can manifest. The reduced radiation dose reflected in the diffraction patterns during this data-collection approach therefore enables the study of micro- and nanometre-sized protein crystals at room temperature under physiological conditions, and has broadened the realm of time-resolved crystallography experiments where reactions can be triggered by light or rapid mixing (Stagno *et al.*, 2016 ▸; Kupitz *et al.*, 2017 ▸; Botha & Fromme, 2023 ▸). The femtosecond X-ray pulses further enable the study of radiation-sensitive systems (Barty *et al.*, 2012 ▸). In a standard serial femtosecond crystallography (SFX) experiment, the millions of individual crystals are streamed through the X-ray beam in a liquid jet (Weierstall *et al.*, 2012 ▸), while a data frame is collected synchronously with every XFEL pulse arriving at the interaction region. The first-generation detectors featured only one gain setting and their repetition rates were limited to 30 Hz, *e.g.* the Rayonix detector. The first custom-design multiple gain mode detector for use at an XFEL was the Cornell–SLAC Pixel Array Detector (CSPAD) operating at 120 Hz (Blaj *et al.*, 2015 ▸).

As X-ray flux and repetition rates increased, a new generation of detectors was developed (Henrich *et al.*, 2011 ▸; van Driel *et al.*, 2020 ▸) to accommodate higher repetition rates and feature new pixel-by-pixel gain-switching modes, thereby dramatically increasing the dynamic range of the detector. However, these segmented dynamic gain-switching detectors are custom designed by/for their respective X-ray facilities and can pose a challenge for data-analysis personnel in a variety of ways, including but not limited to non-linearity of the intensities in the gain-switching mode, delays in the recording of the per-pixel gain switching and challenges of the per-pixel background correction in the different gain modes; correctly calibrating the detectors is a research project in itself. Mis­calibration of pedestal values or gain-switching regions and parameters can result in pixels or detector panels recording unreliable intensity values, which affects the accuracy of downstream data analysis for both SFX and single-particle imaging experiments. Furthermore, no two SFX experiments are alike since the scattering background on the detector varies depending on how the sample is introduced into the X-ray beam, the X-ray energy, and various unwanted experimental artefacts such as salt deposits on the nozzle, phase changes of the sample carrier medium *etc*. In addition, depending on the scientific case, the signal of interest may vary from single-particle-diffraction large-unit-cell protein crystals to small-unit-cell metallic organic frameworks or even solution scattering. Data analysis during an XFEL experiment therefore usually requires experienced data scientists to flexibly implement hit-finding algorithms or eliminate frames with unwanted artefacts on the fly. To address this challenge, we developed the *Pixel Anomaly Detector Tool* (*PADT*), a user-friendly graphical user interface (GUI) to assist even the novice student with these tasks, improving the accuracy and reliability of diffraction intensity measurements without needing to write custom code. Using machine-learning (ML) algorithms from the *scikit-learn* toolbox (Pedregosa *et al.*, 2011 ▸), such as logistic regression, *K*-nearest neighbours, and decision-tree and random-forest classifiers, *PADT* allows the user to test and train models by classifying detector images according to the problem or task at hand. The tool is written in Python (van Rossum & Drake, 2011 ▸), is highly modular, and can easily be expanded to support other X-ray detectors and file formats to enhance the reliability of XFEL data and streamline the data-analysis process.

Deep learning (LeCun *et al.*, 2015 ▸; Goodfellow *et al.*, 2016 ▸) has become a powerful tool for ML to leverage existing knowledge to present previously annotated data (the ‘training set’) and is therefore becoming increasingly popular across various scientific disciplines. The possibility of performing a wide variety of cognitive and inference tasks after being ‘trained’ successfully makes it particularly powerful in image-processing applications (Géron, 2017 ▸). One of the first applications of deep learning to serial macromolecular crystallographic data was implemented by Ke *et al.* (2018 ▸), where a convolutional neural network to detect Bragg spots was executed to classify crystal ‘hits’ from ‘misses’ and ‘maybes’ for a variety of different proteins and experimental conditions. The authors concluded that a successful outcome is heavily dependent on a strong training set for a particular protein and set of experimental parameters, unfortunately thereby hindering generalizations. A potential solution to this hurdle was implemented by Souza *et al.* (2019 ▸), who introduced a method for generating labelled (desirable and undesirable) diffraction images. The technique produces and labels images via a simulator that receives the properties of the incident X-ray beam, the environment and the structure to be analysed as its input. It thereby generates a synthetic training set of diffraction images with an annotation that is 100% accurate, as opposed to erroneous manually annotated real images. Using their simulated dataset (termed DiffraNet), the authors explored several computer-vision-approach off-the-shelf AutoML optimization tools and found that the best model achieved 98.5% accuracy on synthetic images compared with 94.51% accuracy on real images. A similar approach employing neural networks was investigated by Sullivan *et al.* (2019 ▸) for macromolecular neutron crystallography, a complementary structure-determination technique to X-ray crystallography for determining the positions of low-*z* elements (usually hydrogen). They simulated 100 000 training peaks and demonstrated how ML can be used to refine peak locations and peak shapes, and ultimately yield more accurate integrated intensities for Bragg spots. These ML approaches employing neural networks are not only limited to macromolecular crystallography but also find use in small-molecule crystallography (Oviedo *et al.*, 2018 ▸). Although this field is still in its infancy, the method has been successfully demonstrated on simulated data, but still holds clear bottlenecks when extrapolating to real data. Yet, it contains enormous potential for standardizing serial crystallography data collection and processing, as well as achieving higher accuracy for intensity prediction of Bragg reflections.

Our tool is designed to work on real data, regardless of the problem identified during the course of the experiment (*i.e.* hit finding, background fluctuations, detector artefacts *etc*.), while being executable without any programming knowledge or ML expertise. In this article, we will outline the architecture and principle of *PADT*, and then introduce a test case where the tool was used to improve data-collection statistics of a dataset containing detector-calibration artefacts. *PADT* is open source for academic use through an MIT License. The full package can be downloaded from https://github.com/gihankaushyal/PixelAnomalyDetectorTool, along with installation instructions, a user manual and tutorial data.

## 2. The *Pixel Anomaly Detection Tool* (*PADT*)

### 2.1. Overview

**Figure 1:**
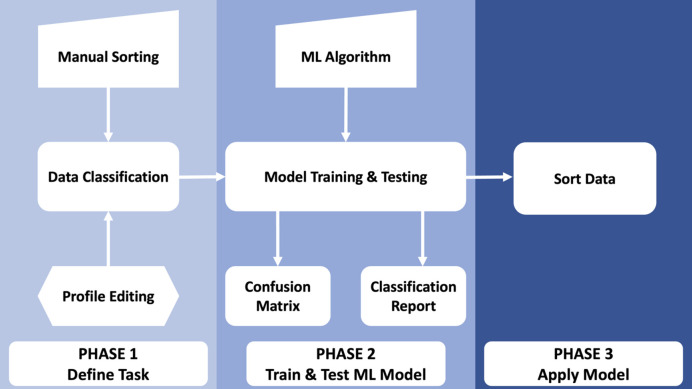
A workflow diagram of the three phases of *PADT*.

*PADT* provides a user-friendly interface that enables users to perform three key phases of image analysis, as shown in Fig. 1 ▸: task definition, model training and testing, and image sorting. All code is written in Python3 (van Rossum & Drake, 2011 ▸) and makes use of the packages *h5py*, *matplotlib*, *numpy*, *Pandas*, *plotly*, *psutil*, *pyqtgraph*, *PyQt5*, *scikit-learn*, *seaborn* and *tqdm*. A full list of prerequisites, tested version numbers and references can be found in Table 1 ▸.

**Figure 2:**
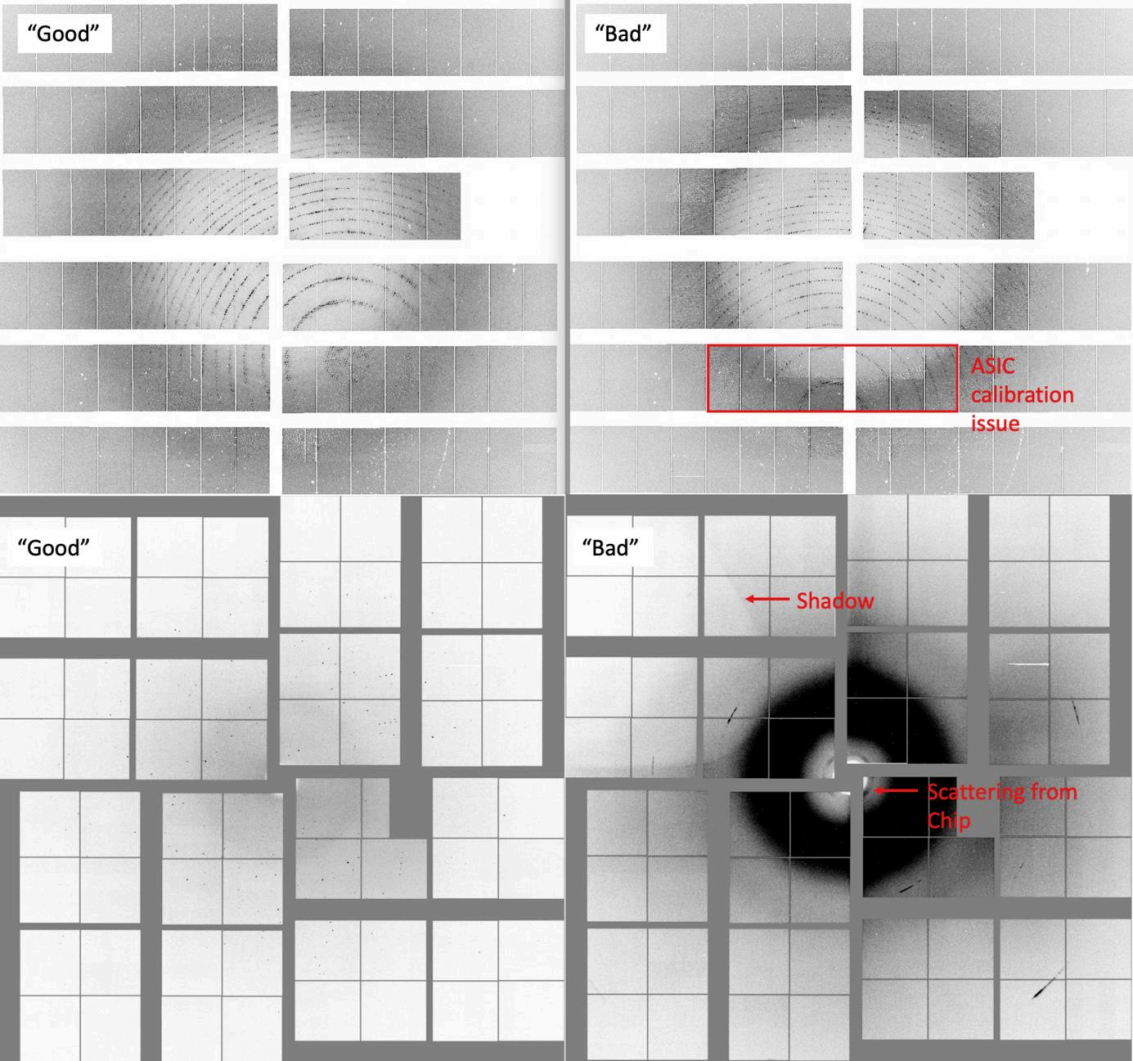
Examples of *PADT* usage cases. ASIC calibration artefacts visible in some diffraction patterns (top). Fixed-target data collection where some images were collected with sub-optimal alignment of the X-ray beam and the sample window (bottom).

During the first phase of *PADT*, the sorting criteria and associated task are defined by the user. A subset of image files are loaded into the GUI and inspected through an image viewer. The user can define what constitutes a ‘good’ image versus a ‘bad’ image depending on the task at hand, either by manually clicking through images in the viewer and selecting the appropriate check box depending on the displayed image or by fitting an expected intensity profile to an area of the detector. In addition to the test case presented in detail below, further examples of good versus bad images that are applicable to *PADT* are shown in Fig. 2 ▸. The top panel shows AGIPD detector frames collected at the European XFEL with (bad) and without (good) an application-specific integrated circuit (ASIC) calibration artefact. The bottom panel of images shows a good image collected using a fixed-target setup and a bad image where the X-ray interaction region is mis­aligned with the sample window, resulting in shadows and parasitic scattering. Both scenarios are handled identically in *PADT*, since it is trained on the fly by the user on the basis of the data, without making any prior assumptions. A 1D vertical projection is displayed alongside each image for the region of interest (ROI) selected, which can aid the user when determining whether profile fitting may be appropriate for pre-classification. When choosing to employ profile fitting, the user can select the order of polynomial to fit, and the GUI will sort training/test image sets according to the location of the inflection points of the fit. The latter method for image classification is particularly useful when there are drastic intensity changes caused by intermittent shadowing or gain-switching artefacts and negates the effort associated with manually clicking through hundreds of images to assemble a reasonably sized training set for phase 2. In the second phase, users can train and test an ML model to take over the large-scale sorting of images. The annotated images from phase 1 are split into training and testing subsets; the user can specify the split fractions, but the default is 70/30 and the GUI will display an error if the split does not equate to 100% (avoiding the potential for unintentional model bias). A range of ML models are supported, as some may perform better than others depending on the task at hand. *PADT* currently supports logistic regression (Cox, 1958 ▸), *K*-nearest neighbours (Fix & Hodges, 1989 ▸; Bentley, 1975 ▸), decision-tree classifier (Quinlan, 1986 ▸; Wu *et al.*, 2008 ▸) and random-forest classifier (Breiman, 2001 ▸; Ho, 1995 ▸). Regardless of the model selected, a confusion matrix and classification reports will be displayed after training and testing are completed, so that the quality of the model can be gauged. In the final phase, the model is applied to data to perform the task it has been trained to do.

### 2.2. The *PADT* GUI

**Figure 3:**
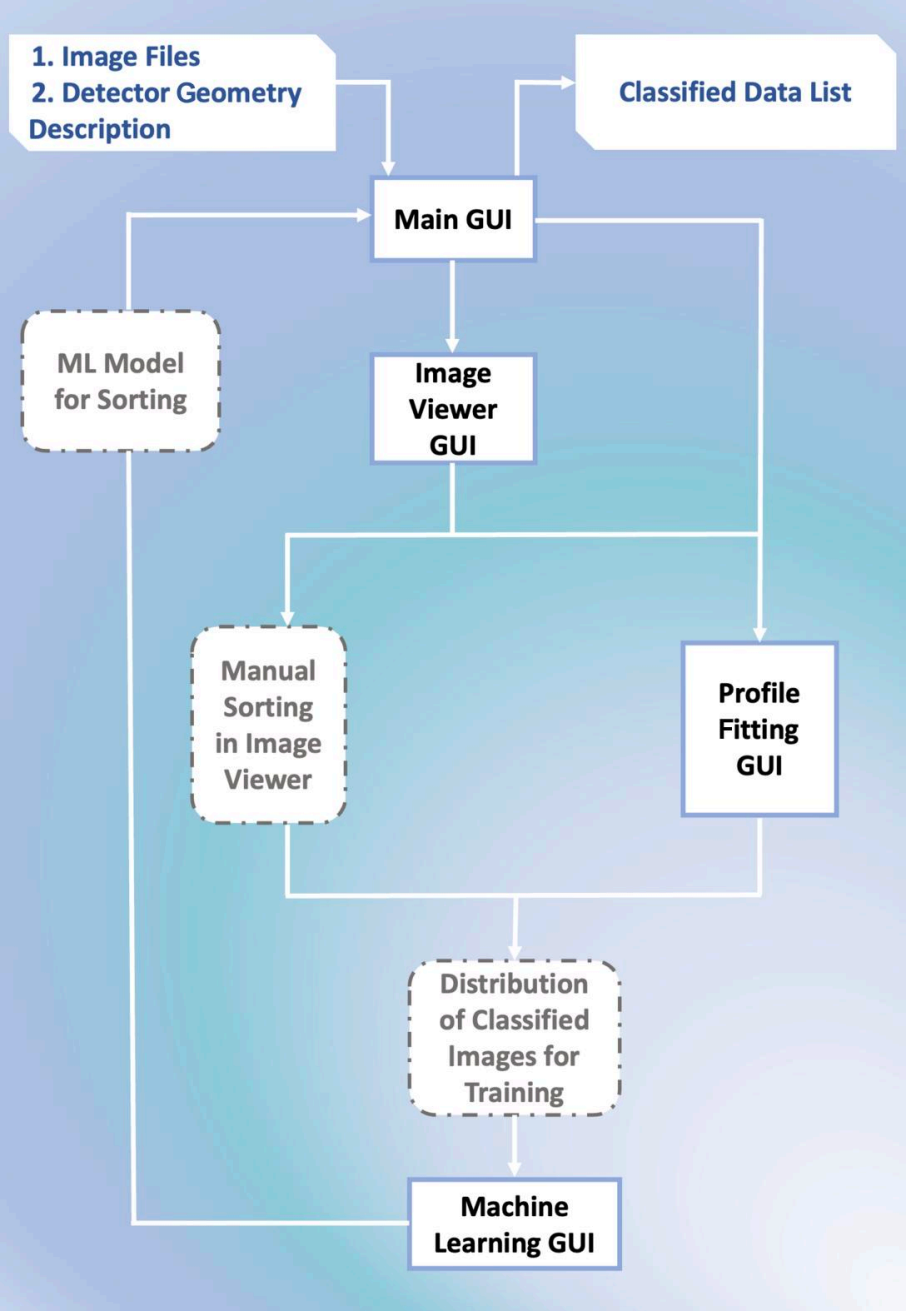
The *PADT* GUI layout. The black boxes refer to physical GUIs included in *PADT*, whereas the dashed grey boxes refer to alternative GUI actions and *PADT* outputs.

The *PADT* GUI is designed to guide the user through the process of assembling the training data, training and testing the model, and then applying it to experimental data. The general workflow is outlined in Fig. 3 ▸. The main GUI is the starting point and successively launches other GUIs as required. Each step is outlined in more detail below.

**Figure 4:**
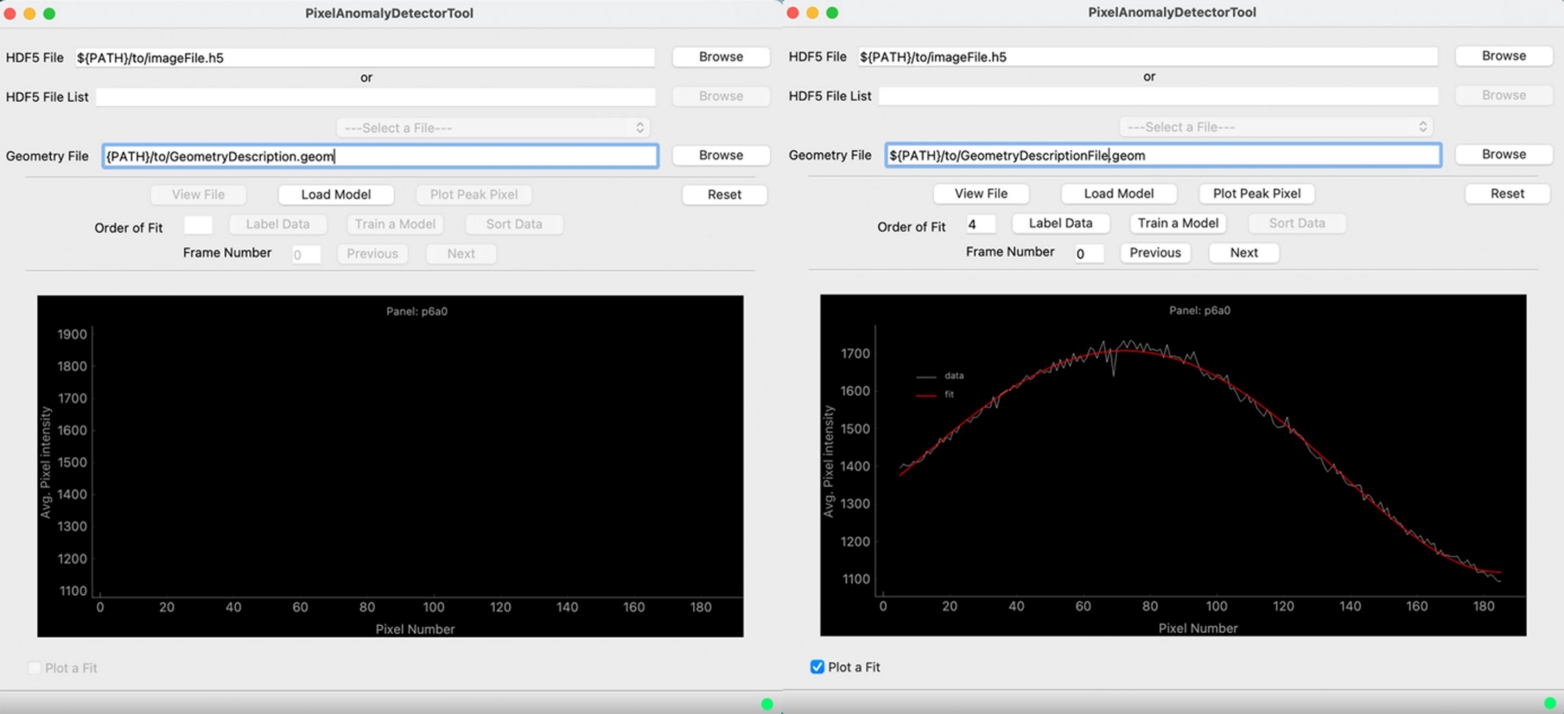
The main window of the tool. When initially launched, the GUI allows limited operations with inactive buttons greyed out (left). As the user is guided through the three phases of the *PADT* process, buttons progressively become active (right). Also displayed is a 1D vertical projection of the selected ROI, along with a fourth-order polynomial fit.

The main GUI is launched when starting *PADT*, with most functionality greyed out and inactive (Fig. 4 ▸, left). As the user traverses the different phases of *PADT*, the buttons successively become active, guiding the user through the process (Fig. 4 ▸, right). *PADT* supports the input of multi-event HDF5 image files (Collette, 2013 ▸), currently the most common format of detector image files at XFELs, or a list of multiple HDF5 files. Since many XFELs use segmented detectors, *PADT* also requires a detector-geometry description in *CrystFEL* (White *et al.*, 2012 ▸) format to display the images in the laboratory coordinate frame.

**Figure 5:**
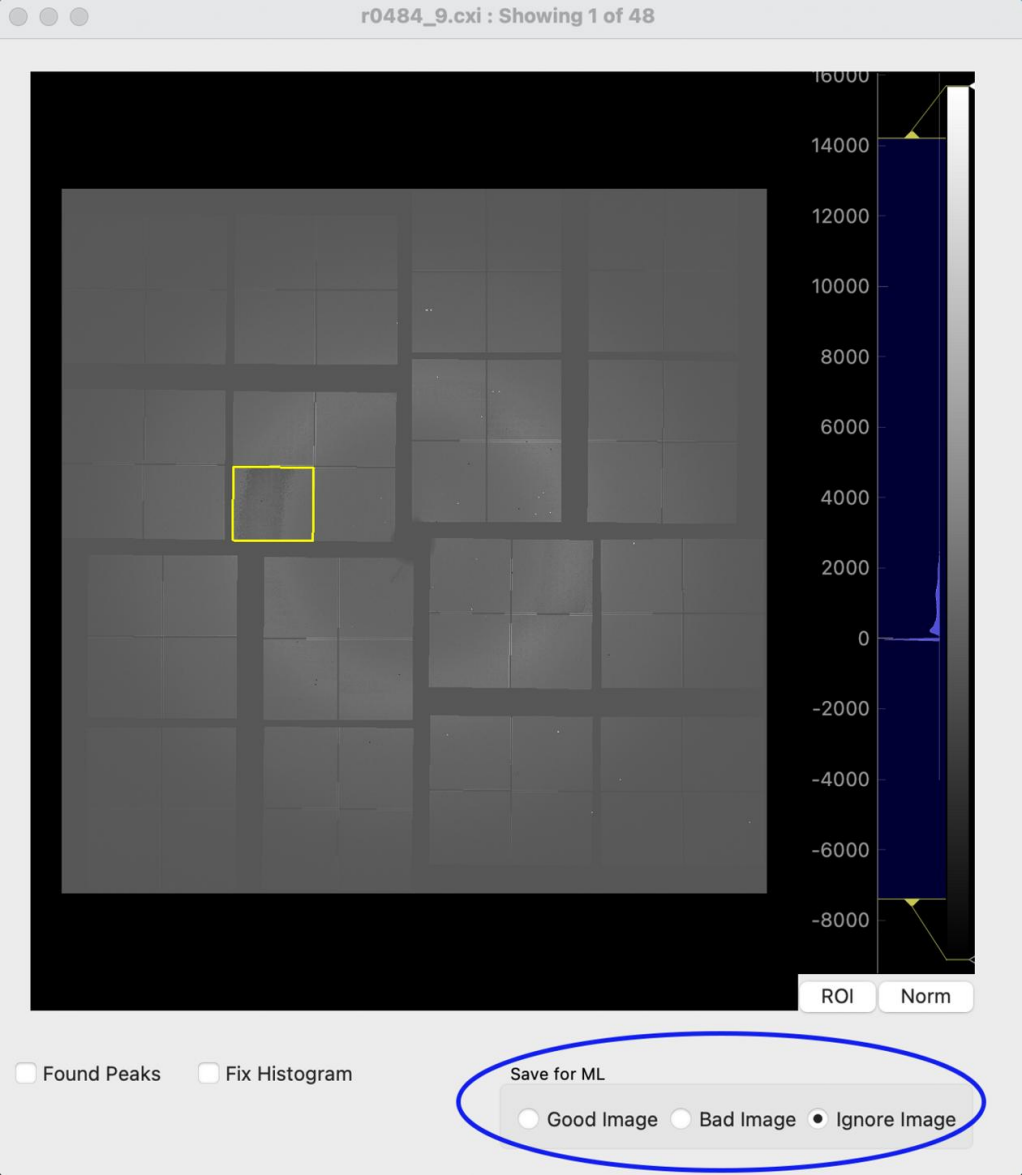
Manual sorting within the *PADT* image-viewer GUI. By selecting the appropriate check box, an image can be added to the training dataset as being good or bad, or if the user is unsure the image can be skipped. The image loaded into the GUI shows a non-linearity particularly pertinent in the ROI selected (yellow box). The viewer itself is adapted from the *Cheetah* image viewer *cxiview* (Barty *et al.*, 2014 ▸), with all functionality, along with the *PADT* sorting ability.

The image viewer is launched directly from the main GUI (‘View File’) and is a modified and fully integrated version of the *Cheetah* image viewer *cxiview* (Barty *et al.*, 2014 ▸). Interactively clicking the mouse on a detector panel in the viewer (Fig. 5 ▸) will automatically select this panel as the ROI for ongoing analysis. Additionally, a 1D vertical projection of the pixel intensity values for the ROI can be displayed in the main GUI (Fig. 4 ▸, right). Optional further functionality includes fitting a polynomial to the 1D intensity profile and using the location of the inflection points to automatically sort the data. This is particularly useful when there is gross intensity variation between good and bad images or when a known scattering profile is desired (*e.g.* during solution scattering experiments). Alternatively, and particularly useful for the inexperienced scientist, images can also be directly tagged through the image viewer.

This allows the user the utmost flexibility of classifying data according to arbitrary criteria dictated by the task at hand while using real data. The disadvantage of this approach is the speed with which a human can tag sufficient data during an ongoing experiment to assemble a large enough training set for establishing a robust and reliable model. Regardless of the chosen method for assembling the training data, *PADT* outputs two text files containing lists of the image file names (and event numbers for multi-event files, if applicable) for the good and bad image files. Therefore, should an interruption occur, the task can be resumed and does not need to be re-started. Alternatively, multiple instances can be sorted in parallel by multiple users to speed up the process of tagging the training data, and then concatenated into a single folder for input into the ML training and testing GUI. Good and bad are simple descriptors and both datasets are handled identically (*i.e.* *PADT* will sort into two distinct datasets, either of which can be subjected to downstream processing).

**Figure 6:**
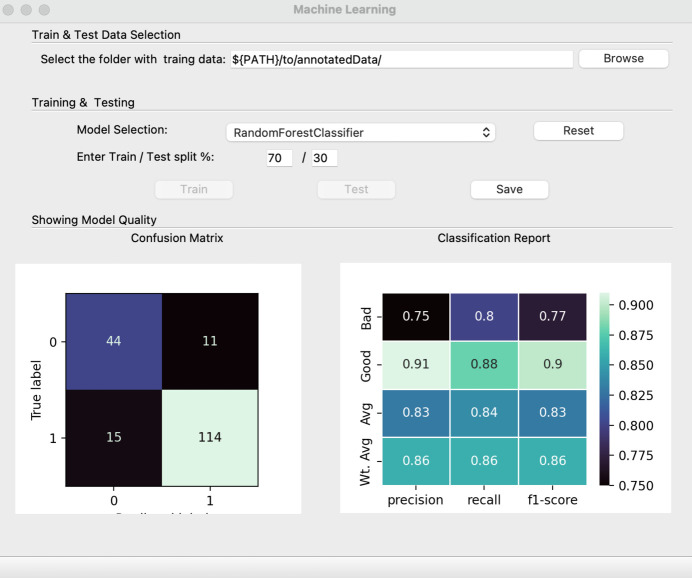
The model training and testing GUI. Once training and testing are completed on the training dataset, the confusion matrix and classification report can be inspected.

Once satisfied with the training dataset, the user can launch the ML model training GUI (Fig. 6 ▸) from the main GUI via the ‘Train a Model’ button. After pointing the application to the folder containing the annotated data, the user has the option to select from four different integrated ML algorithms, which are detailed in the next section. The train/test data split can be further adapted if required. The split is implemented randomly across the pre-classified dataset and the test set is excluded during model training to avoid bias.

**Figure 7:**
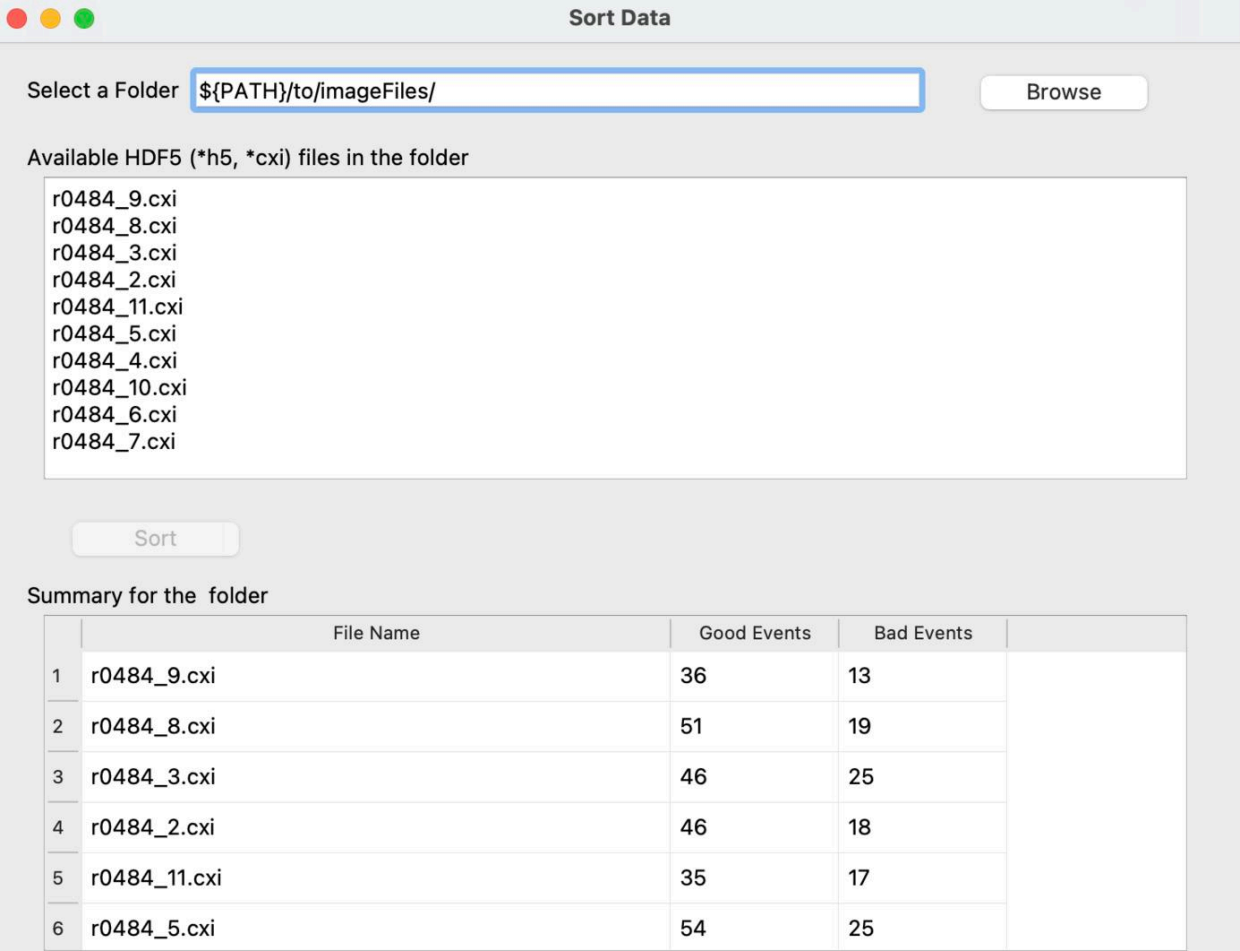
The data-sorting GUI. *PADT* can now be pointed at any dataset and will automatically use the most recently trained ML model to sort the data for further analysis.

The estimated model quality is displayed directly in the GUI in the form of a confusion matrix and classification report, the interpretation of which is elaborated on below. Once a satisfactory model has been obtained, it can be saved for future use. The trained and tested model can now flexibly be loaded through the main GUI to sort a dataset at any point in time. Selecting the ‘Sort Data’ button will launch the sorting GUI (Fig. 7 ▸) with the model pre-loaded. *PADT* can now be pointed towards a folder containing multi-event HDF5 files for sorting, and will write a list with good and bad events as well as displaying a summary for each file in the GUI.

### 2.3. Additional *PADT* features

While *PADT* is constantly being improved, some features that are particularly beneficial towards the ‘user friendliness’ aspect already implemented are outlined below:

(*a*) Status bar: the *PADT* GUI is designed to provide an exceptional user experience and effortless navigation. Whenever users interact with the tool, they receive helpful messages on the status bar that serve as navigational aids, providing real-time feedback and guidance for optimal usage.

(*b*) Tooltip: to help users understand the tool’s functionality and use it effectively, each interactive button on the *PADT* GUI has a tooltip that provides a brief description of its function and purpose. This ensures that users can harness the full potential of the tool without any confusion.

(*c*) Error messages: if there are any operational issues or user errors, the *PADT* GUI uses dialogue boxes to display error messages promptly. This proactive approach ensures that users are informed of any problems and can take corrective measures with confidence.

(*d*) Parallelization: from a technical perspective, *PADT* is highly adaptable. It is fully compatible with message passing interface (MPI) parallelization systems. Furthermore, if a system does not support MPIs, *PADT* can leverage multiple processes through multi-threading. If neither MPI nor multi-threading is feasible, the tool can still operate in a single-threaded mode. This multi-tiered approach guarantees optimal performance across different system architectures.

### 2.4. *PADT* ML algorithms

Machine-learning algorithms are a subset of artificial intelligence that enable computers to learn and make predictions or decisions without being explicitly programmed. In the context of *PADT*, these algorithms are used to classify and subsequently sort detector frames as good or bad depending on the problem definition.

*PADT* currently supports several ML algorithms, including logistic regression (Cox, 1958 ▸), *K*-nearest neighbours (Bentley, 1975 ▸; Fix & Hodges, 1989 ▸), decision-tree classifier (Quinlan, 1986 ▸; Wu *et al.*, 2008 ▸) and random-forest classifier (Ho, 1995 ▸; Breiman, 2001 ▸). All of the ML algorithms implemented in *PADT* are part of the *scikit-learn* library (Pedregosa *et al.*, 2011 ▸). Here is a brief explanation of how each algorithm works:

(i) Logistic regression: this is a binary classification algorithm that uses a logistic function to model the probability of a certain class. It tries to find the best decision boundary that separates the classes in the input data (sklearn.liner_model.LogisticRegression).

(ii) *K*-nearest neighbours: this is a non-parametric classification algorithm that uses a distance metric to find the *K*-nearest training examples to a given test example. The class of the test example is then determined by a majority vote of the *K*-nearest neighbours (sklearn.neighbours.KNeighbors).

(iii) Decision-tree classifier: a decision tree is a hierarchical model that uses a set of if–then rules to make decisions. A decision-tree classifier works by recursively partitioning the input space into subsets based on the values of different features in the input data. At each node in the tree, a decision is made according to the value of a particular feature, and the process continues until a leaf node is reached that corresponds to a particular class (sklearn.tree.DecisionTreeClassifier).

(iv) Random-forest classifier: random forest is an ensemble learning method that combines multiple decision trees to improve the accuracy of the classification. The algorithm works by building a set of decision trees on random subsets of the input data and random subsets of the features. The final classification is then determined by a majority vote of the individual trees (sklearn.RandomForestClassifier).

In the context of *PADT*, the ML algorithms are trained on a set of diffraction images with known labels of good or bad. The input data for each algorithm consist of the pixel intensity values for the ROI selected for each diffraction image. The algorithms are then evaluated on a separate set of test images to determine their accuracy in classifying the images into two distinct datasets. The user can select the best-performing algorithm for the task at hand before moving on to large-scale image sorting.

Overall, the ML algorithms in *PADT* are a powerful tool for automating the process of flexibly classifying diffraction images, saving valuable time and effort for data-analysis personnel.

### 2.5. Model quality diagnostics

In the realm of ML, evaluating the efficacy and precision of a model is of paramount importance. Two pivotal evaluation metrics that stand out in this context are the confusion matrix and the classification report. Both serve as critical tools to assess and refine the performance of ML models, albeit in slightly different ways.

The confusion matrix, as its name implies, lays out a matrix-form representation of a model’s predictions against the actual class labels. Annotated data that were excluded from model training (*i.e.* the ‘test’ fraction) are sorted by the model and the outcome is compared with the assigned labels for compliance. The visual representation is divided into four main components: true positives (TP), true negatives (TN), false positives (FP) and false negatives (FN). By presenting these values, the matrix offers insights into the number of correct predictions made for each class, as well as those that were classified incorrectly from the assigned label. The power of the confusion matrix lies not only in its ability to give an overall view of the model’s prediction accuracy but also in highlighting specific areas where the model might be faltering. This makes it an invaluable tool for data scientists and ML engineers, as it helps them recognize patterns, potential biases and areas of improvement for their models. Equations (1) and (2), shown below, allow calculation of the accuracy and mis­classification rate to evaluate the performance of a classification model, considering the TP, TN, FP and FN obtained from the confusion matrix: 

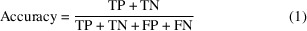

and 

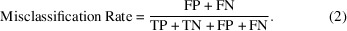




The classification report, on the other hand, dives deeper, offering a more granular view of the model’s performance. Rather than just showing prediction outcomes, it calculates and presents several vital metrics, giving a more rounded perspective of the model’s efficacy. These metrics include the following:

(*a*) Precision quantifies how many of the predicted positive instances are actually positive:

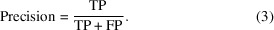




(*b*) Recall (or sensitivity) gauges the model’s capability to identify all positive instances correctly:

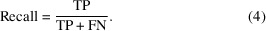




(*c*) F1 score is the harmonic mean of precision and recall. The F1 score provides a balance between the two metrics, especially useful when the class distribution is uneven:

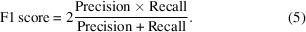




(*d*) Support represents the number of actual occurrences of the class in the dataset, giving context to the other metrics: 

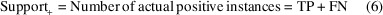

and 

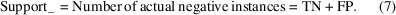




The classification report therefore paints a detailed picture of a model’s strengths and weaknesses. The presented metrics elucidate the model’s efficiency in making correct predictions, its rate of FP, the balance between precision and recall, and more.

## 3. Example case

The efficacy of *PADT* was tested on a subset of SFX data that has been published previously. This particular dataset was chosen since it had necessitated elaborate data-analysis efforts to mitigate the effects of a gain-switching artefact on the detector prior to publication. Specifically, testing was performed on SFX data from the SARS-CoV-2 NendoU protein (Jernigan *et al.*, 2023 ▸). The data were collected in 2021 using the ePix10k-2M detector (van Driel *et al.*, 2020 ▸) at the macromolecular femtosecond crystallography instrument (Sierra *et al.*, 2019 ▸) at the Linac Coherent Light Source in California. For more experimental data-collection details, please refer to Jernigan *et al.* (2023 ▸), as they are not relevant for demonstrating the functionality of *PADT*. Of importance, and why this particular dataset was chosen, are the effects of the gain-switching artefact resulting in miscalibrated intensities when the diffuse scattering from the water ring falls into a critical intensity range. An adversely affected panel is selected as the ROI in Fig. 5 ▸, and preparing these data for publication required extensive data-analysis efforts at the time. In Fig. 9 it can be seen how the miscalibration features extend for an entire *q* range for data collected at this critical intensity threshold.

Thirty random multi-event HDF5 files containing SFX crystal diffraction patterns (post-hit finding) were selected (2606 images in total) and annotated using both the profile-fitting approach and manual sorting for the model training set. An unanticipated hurdle encountered during the profile-fitting approach was that *PADT* assumes that the majority of image files constitute good data and therefore suggests that the inflection points located within the dominant histograms are good images. This may not always be the case depending on the subset, so special attention needs to be paid whenever concatenating the training dataset out of multiple separate instances of *PADT* profile-fitting classification runs. To ensure a trusted and verified training set, the 2606 images were sorted manually using the viewer, with 790 images being classified as good and 1816 as bad for the training dataset. In preliminary tests it was determined that at least 2000 images are required for reliable model training and testing for this particular case. At this point, no attention was paid to the quality or potential indexability of the crystal diffraction patterns.

The training dataset was applied to all four of the ML algorithms and the respective models calculated and saved. The default split of 70/30 training/testing ratio was used. The saved models were then used on a larger portion of data collected under the same experimental conditions (104 731 images in total), while special attention was paid to not include any of the images used to train and test the model in the datasets sorted with the trained models and reported below. The sorting accuracy was evaluated for each sorting algorithm by manually inspecting the sorted results for ∼2200 random images and verifying whether they had been correctly sorted as good images (TP), bad images (TN) or neither (FP and FN). The results from this assessment for the four supported ML algorithms are presented in Table 2 ▸.

From this comparison, the random-forest model most accurately identified good images (*i.e.* TP) as well as bad images (*i.e.* TN), making it clearly superior to the other models for this particular case (94.3 and 98.3%, respectively). Since the random-forest model presented the lowest mis­classifying rate overall (5.7 and 1.7% for good and bad datasets, respectively), the impact of applying the model for data classification versus not applying the model (and benefiting from higher data redundancy instead) was further investigated.

**Figure 8:**
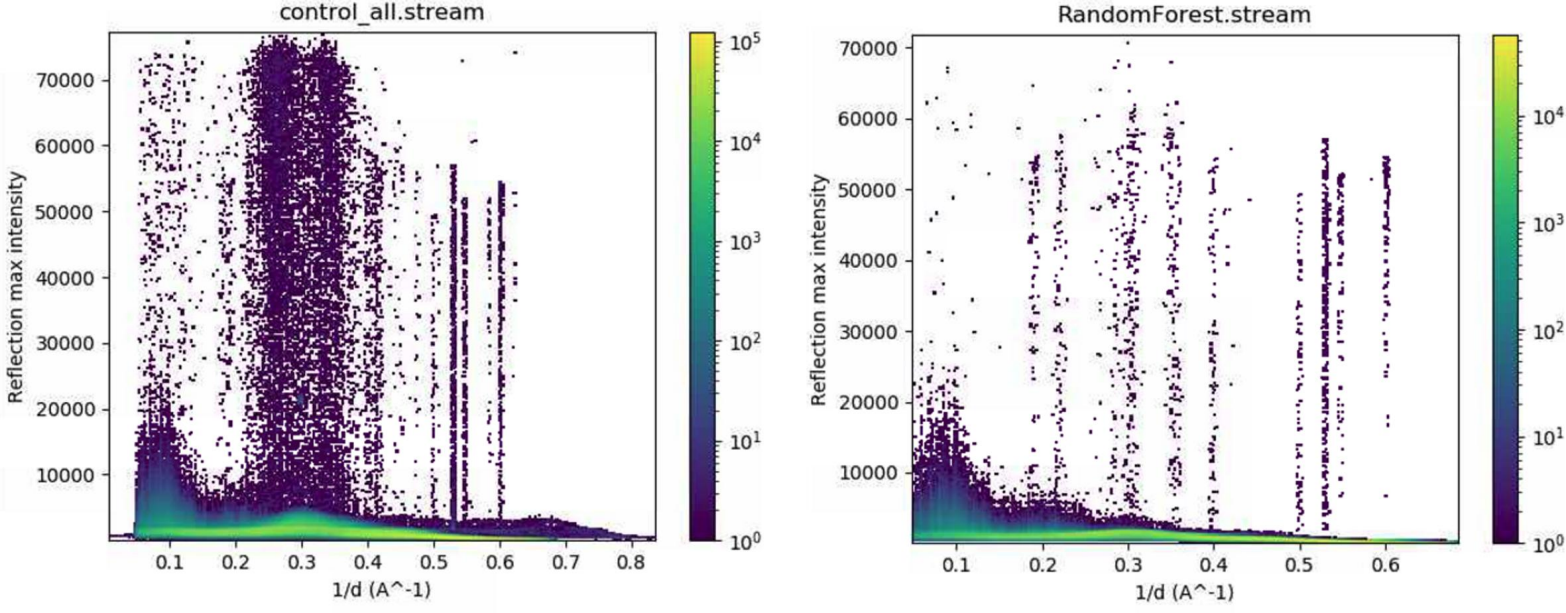
Comparison of the maximum intensity measured for every reflection versus 1/resolution (Å). Control dataset (19 284 indexed images) (left). Random-forest-classified dataset (11 269 indexed images) (right).

General crystallography data-collection statistics were calculated for the full unclassified dataset (104 709 apparent crystal hits), referred to as the ‘control group’, as well as for the 23 979 crystal hits contained in the dataset classified as good by the random-forest model. No new attempt was made to optimize hit-finding or peak-finding parameters; the data were reused as processed on the fly during the beam time. In brief, datasets were submitted to indexing using *CrystFEL* version 0.9.1 (White *et al.*, 2012 ▸), with the following parameters and options: –peaks = cxi; –int-rad = 2,4,6; –multi and –check-peaks. Hence, indexing was performed using the peaks stored in the HDF5 files from the previous processing and not optimized [as they had been by Jernigan *et al.* (2023 ▸) where a second round of hit and peak finding was performed under optimized conditions]. Successive indexing attempts were made using *XDS* (Kabsch, 2010 ▸), *Mosflm* (Powell *et al.*, 2013 ▸), *Dirax* (Duisenberg, 1992 ▸) and *Xgandalf* (Gevorkov *et al.*, 2019 ▸), in that order with unit-cell parameters *a* = 154 Å, *b* = 154 Å, *c* = 117 Å, α = 90°, β = 90° and γ = 120°. The only pixel masks applied to the detector images were the bad pixel mask from the beam time (containing hot and dead pixels) and the panel edges; no further mask refinement was implemented on the basis of data-processing results. Under these conditions, 19 284 images were successfully indexed for the all-encompassing control dataset (18%) and 11 269 images for the random-forest-classified dataset (47%). Interestingly, the substantially higher indexing rate for the random-forest dataset clearly indicates that the hit and peak finding carried out during the experiment were considerably hindered by the erroneous regions of the detector. The maximum intensity integrated for every reflection is plotted against resolution in Fig. 8 ▸ for the random-forest good dataset (right) as well as the complete all-encompassing dataset (left).

The higher occurrence of over-estimated intensity values, particularly in the region most commonly effected by gain-switching errors (0.2–0.4 Å^−1^; 5.0–2.5 Å), is evident, indicating that many of the indexed hits from the control dataset still contain images with miscalibrated pixel values. The indexing results for both datasets were then merged into point group 622 using *CrystFEL*’s *partialator* program (White *et al.*, 2012 ▸) with the following settings: unity model, 1 iteration.

**Figure 9:**
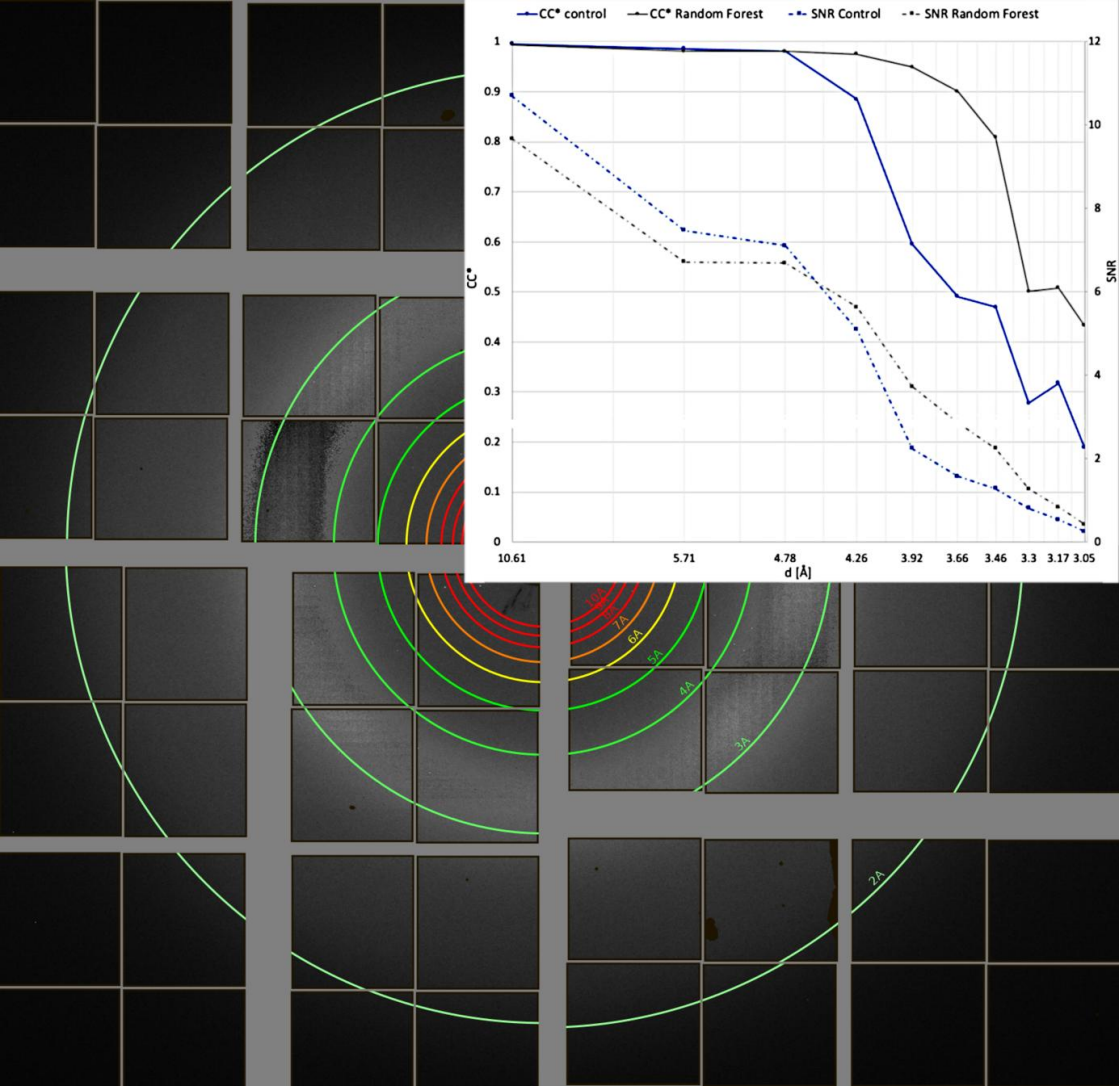
A diffraction pattern with anomalous detector behaviour in the range 5.0–2.5 Å. Inset: SNR and CC* calculated from the merged intensities in different resolution shells for the control and random-forest datasets.

The signal-to-noise ratio (SNR) as well as CC*, a statistic commonly reported for crystallography datasets that estimates the correlation of an observed dataset with the underlying true signal (Karplus & Diederichs, 2012 ▸), are plotted against resolution for both merged datasets in Fig. 9 ▸. Interestingly, the random-forest dataset has both lower SNR and marginally lower CC* values at lower resolution. However, in the region where the pixels display the anomalous behaviour, the substantially smaller random-forest dataset notably outperforms the control dataset. This is reflective of the higher redundancy of the control dataset beneficially impacting the statistics at lower resolution, but the inclusion of bad data results in inconsistent intensity measurements at higher resolution that are outweighed by the inclusion of more data. This example case demonstrates how *PADT* can easily aid in improving crystallographic data statistics during an ongoing experiment or after, without the need for writing a single line of code.

## 4. Conclusions

Here we introduced a user-friendly GUI interface for image classification, which is particularly amendable to data collected at an XFEL. Based on ML algorithms and a supervised learning approach, *PADT* supports a wide range of data-classification and -analysis tasks without the need to write code for the specific task at hand. While *PADT* currently supports the Epix2k-4M (van Driel *et al.*, 2020 ▸) detectors and file formats at the LCLS as well as the AGIPD detector (Henrich *et al.*, 2011 ▸) at the European XFEL GmbH (Altarelli *et al.*, 2006 ▸), we are working on making it universally adaptable to any X-ray detector. In particular, unsegmented detectors, the norm at synchrotrons, will soon also be supported. Currently, the specification of an ROI is necessary to maintain reasonable processing speeds, but implementing HPC support will make this requirement obsolete in the future. Furthermore, an in-GUI peak finder to aid in on-the-fly hit finding during training will make manual image selection for hit finding obsolete. In summary, the applications that can benefit from *PADT* extend far beyond XFEL data analysis. 

## Figures and Tables

**Table 1:** A list of prerequisites and version numbers tested for functionality (in alphabetical order)

Package	Version	Reference
Python	3.8	van Rossum & Drake (2011 ▸)
*h5py*	0.8.8	https://www.h5py.org/
*matplotlib*	3.5.2	Hunter (2007 ▸)
*numpy*	1.23.3	Harris *et al.* (2020 ▸)
*Pandas*	1.4.4	Pandas Development Team (2020 ▸)
*plotly*	5.9.0	https://plot.ly
*psutil*	5.9.0	https://github.com/giampaolo/psutil#readme
*pyqtgraph*	0.12.3	https://www.pyqtgraph.org
*PyQt5*	5.15.16	*PyQt* (2012 ▸)
*seaborn*	0.11.2	Waskom (2021 ▸)
*scitkit-learn*	1.1.3	Pedregosa *et al.* (2011 ▸)
*tqdm*	4.64.1	da Costa-Luis (2019 ▸)

**Table 2:** Performance comparison for ML algorithms for example data verified by manually inspecting 2200 random images from the final sorted dataset after applying the respective models

	Logistic regression	*K*-nearest neighbour	Decision tree	Random forest
True positive	91.5%	78.2%	91.4%	94.3%
False positive	8.5%	21.8%	8.6%	5.7%
True negative	97.5%	90.0%	97.4%	98.3%
False negative	2.5%	10.0%	2.6%	1.7%
